# First report of *Penicillium ulaiense* causing whisker mold on postharvest citrus fruits in Morocco

**DOI:** 10.1038/s41598-026-56301-2

**Published:** 2026-06-22

**Authors:** H. Benzahra, I. Mrabti, H. Grijja, R. Azenzem, R. Abou Kubaa, M. Afechtal, K. Selmaoui

**Affiliations:** 1 Laboratory of Botany, Biotechnology and Plant Protection, Faculty of Sciences, University Ibn Toufail, Kenitra, Morocco; 2 Laboratory of Virology, National Institute of Agricultural Research, 14 rue Ibn Temmam, PO Box 257, 14000 Kenitra, Morocco; 3 Department of Plant Pathology, University of California, Davis, CA 95616 USA; 4 Research Unit on Nuclear Techniques, Environment and Quality, Regional Centre of Agricultural Research of Tangier, National Institute of Agricultural Research, Avenue Ennasr, BP 415 Rabat Principale, 10090 Rabat, Morocco

**Keywords:** Citrus postharvest disease, *Penicillium ulaiense*, Morphological identification, DNA barcoding, ITS, β-tubulin, Microbiology, Plant sciences

## Abstract

Between February 2024 and June 2025, surveys conducted in northwestern Morocco revealed citrus fruits (*Citrus sinensis* and *Citrus × limon*) showing atypical postharvest decay symptoms, characterized by dark, water-soaked lesions with zonation and coremia-like structures. These symptoms were detected at only two sites, with a low incidence of 1.6% in the orchard and 0.5% in market samples. Five fungal isolates were obtained from symptomatic tissues on potato dextrose agar (PDA). Morphological characterization identified all isolates as *Penicillium ulaiense*, based on colony and micromorphological features consistent with published descriptions. Two representative isolates (BH_B1 and BH_E2) were selected for molecular analyses. Sequencing of the ITS region and the β-tubulin (BenA) gene showed 99–100% identity with reference *P. ulaiense* sequences, and the concatenated alignment comprised 804 base pairs. Phylogenetic analyses placed the Moroccan isolates within a well-supported *P. ulaiense* clade, clearly distinct from closely related species such as *P. italicum* and *P. expansum*. Pathogenicity assays reproduced typical symptoms on *C. sinensis* fruits, with progressive lesion development and successful re-isolation of the pathogen, confirming its pathogenic role under experimental conditions. This study reports, for the first time, the occurrence of *Penicillium ulaiense* associated with postharvest whisker mold symptoms on citrus fruits in Morocco. It provides new insights into postharvest citrus pathogens in Morocco and establishes a basis for future investigations on the occurrence and epidemiology of this species.

**Supplementary Information:**

The online version contains supplementary material available at https://doi.org/10.1038/s41598-026-56301-2.

## Introduction

Citrus production constitutes a strategic pillar of Morocco’s agricultural economy, significantly contributing to national income, food security, and rural employment^1,2^. This sector benefits from particularly favorable agro-climatic conditions in key regions such as Marrakech-Safi, Souss-Massa, and Gharb, where a temperate climate, high solar exposure, and efficient irrigation systems enable the continuous production of high-quality citrus fruits^1^. Morocco is internationally recognized for its high-value citrus varieties, including Navel oranges, clementines, and lemons, which are widely exported to Europe, Russia, and the Middle East^1,3–7^. According to the United States Department of Agriculture Foreign Agricultural Service^8^, Moroccan citrus exports are projected to reach 597,000 tonnes during the 2024–2025 season, representing a 31% increase compared to the previous season. This trend reflects the country’s growing position in the global market and highlights the increasing need to preserve post-harvest fruit quality under increasingly demanding international market conditions. Despite this favorable dynamic, the sector’s competitiveness remains strongly influenced by post-harvest losses, which constitute a major factor affecting fruit quality and quantity. The post-harvest phase is particularly critical, as fruits are vulnerable to mechanical injuries and fluctuations in temperature and humidity, conditions that favor the development of fungal pathogens^9,10^. Several fungal genera are regularly associated with citrus decay, including *Colletotrichum*, *Geotrichum*, *Alternaria*, *Phytophthora*, and *Penicillium*, causing diseases such as anthracnose, sour rot, black rot, or brown rot, which significantly compromise fruit quality, shelf-life, and commercial value^11–13^. 

Among these genera, *Penicillium* plays a predominant role due to its frequency and the extent of losses it causes. Globally, three main species are responsible for post-harvest citrus rots: *P. digitatum* (green mold), *P. italicum* (blue mold), and *P. ulaiense* (black mold)^12,14–16^. In contrast to *P. digitatum*, which produces soft lesions covered with dense olive-green sporulation, and *P. italicum*, characterized by wet lesions with abundant bluish-green sporulation, *P. ulaiense* is distinguished by darker, well-defined lesions associated with grayish-blue to black sporulation and the possible formation of synnemata-like (coremia-like) structures, which represent a key diagnostic feature for distinguishing this species from other *Penicillium* spp. Schoch^17^, Smilanick^16^. Moreover, its relatively slower growth and less conspicuous early symptoms may lead to under-detection during routine inspections, thereby complicating early diagnosis^16^. *P. ulaiense* has been reported in several citrus-producing regions worldwide. In North America, it was first described in California (USA) by Holmes^18^. In Asia, its occurrence has been reported in Japan^19^, Pakistan^20^, and Korea^21^. In Europe, it was later identified in Spain^22^. In Africa, the pathogen has been reported in Tunisia^23^ and Egypt^24^. The increasing number of reports across diverse geographic regions suggests a wider distribution of *P. ulaiense* than previously recognized. However, to date, it has not been reported in Morocco, representing an important gap in current knowledge of post-harvest fungal pathogen diversity in the country.

Despite being less frequently reported than the other two species, *P. ulaiense* may be underestimated in several regions due to its slower growth and subtle early symptoms, which complicate early diagnosis and may lead to under-recognition in surveys.

In Morocco, *P. digitatum* and *P. italicum* are well-established as major post-harvest mold agents. The first report of *P. digitatum* on Moroccan citrus dates back to 1990, while the first record of *P. italicum* was documented in 1995, revealing that fruits are highly susceptible to infection even from minor injuries to the peel^25–27^. These studies also highlighted variations in fungicide sensitivity, emphasizing the need for integrated management strategies tailored to local conditions. However, most available studies have focused primarily on these two species, leaving limited information on other potentially present species and suggesting that the diversity of *Penicillium* spp. associated with Moroccan citrus may be underestimated.

During field surveys conducted between February 2024 and June 2025 in northwestern Morocco, a significant number of fruits exhibited typical symptoms of *P. digitatum* and *P. italicum*, including soft or wet lesions and green or bluish-green sporulation. Concurrently, other fruits consistently displayed atypical lesions, characterized by dark, circumscribed, or water-soaked areas associated with sporulation morphologically distinct from these two common species. These observations suggest the presence of a different or previously uncharacterized pathogen under local conditions. These field observations constitute the starting point of the present study and highlight the need for accurate identification using integrated approaches.

These findings motivated the present study. The main objective is to isolate and identify the causal agent of these atypical symptoms using detailed morphological analyses combined with molecular approaches. Given the limited number of samples analyzed, this study is intended as a preliminary report and does not aim to infer the distribution or prevalence of the pathogen in Morocco. Its primary objective is to document the potential presence of *P. ulaiense* and to provide a preliminary scientific basis for future, more comprehensive investigations.

Furthermore, enhancing knowledge of *Penicillium* diversity in Moroccan citrus remains essential, as current data are still limited. This work provides a first step toward improving the identification of post-harvest pathogens and contributes to a better understanding of their diversity, without extending conclusions beyond the available data.

## Materials and methods

### Sample collection and fungal isolation

Between February 2024 and June 2025, phytopathological surveys were conducted in northwestern Morocco to identify the causal agent responsible for atypical postharvest rot symptoms on citrus fruits. A total of 36 sites were surveyed, including 30 orchards and 6 local markets, covering different stages of the postharvest chain. Surveys were carried out both in experimental orchards, particularly at the INRA El Menzeh station (Kénitra), and in commercial distribution environments. Among these sites, only one orchard (INRA El Menzeh station, Kénitra) and one local market showed fruits exhibiting atypical symptoms corresponding to those investigated in this study. A total of five symptomatic fruits were collected, including four from the INRA El Menzeh research station (Kénitra) and one from a traditional market. The sampled fruits mainly belonged to *Citrus sinensis* and *Citrus × limon*, and corresponded to different stages of the postharvest chain (pre-storage at orchard level and post-storage at market level). In addition to sampling, a disease incidence assessment was conducted across all surveyed sites. In each orchard, 25 trees were randomly selected, and for each tree, 10 leaves, 10 twigs, and 10 fruits were examined for symptom presence. Disease incidence was defined as the percentage of organs showing at least one symptom and was calculated as the ratio of symptomatic organs to the total number of organs observed, multiplied by 100.

The fruits exhibited soft or dark lesions, water-soaked tissues, and unusual sporulation. They were transported to the laboratory under sterile and controlled conditions, and for each sample, the origin, fruit type, and detailed symptom description were recorded. Tissue fragments (5 × 5 mm) excised from the margin between healthy and symptomatic tissues were surface-sterilized in 1% sodium hypochlorite for 1 min, rinsed three times with sterile distilled water, dried on sterile filter paper, and incubated in Petri dishes lined with moistened filter paper at 25 ± 2 °C for 5–7 days in darkness. Emerging fungal colonies were subcultured onto Potato Dextrose Agar (PDA) supplemented with antibiotics to obtain pure cultures, resulting in five fungal isolates showing similar morphology to each other but distinct from *Penicillium* species commonly associated with citrus, such as *P. digitatum* and *P. italicum*. This distinction was based on colony morphological traits, fruit symptom characteristics, and comparison with diagnostic descriptions reported in specialized literature^15,28,29^. The isolates were purified using single-spore cultures (BH_B1, BH_E2, BH_M3, BH_A4, BH_G5) and stored at 4 °C. All isolates were maintained on PDA slants and periodically subcultured to preserve viability and morphological stability.

### Morphological characterization

Fungal isolates (BH_B1, BH_E2, BH_M3, BH_A4, and BH_G5) were cultured on Potato Dextrose Agar (PDA; Merck^®^, Germany) for morphological characterization. Each isolate was grown on three independent Petri dishes, and the experiment was conducted in triplicate to ensure reproducibility. Cultures were incubated at 25 ± 2 °C in the dark under controlled relative humidity (~ 70%), simulating post-harvest conditions. Colony morphology was assessed after 5–7 days based on macroscopic features including texture, pigmentation, growth pattern, and sporulation intensity. Colony diameters (mm) were measured on each Petri dish to estimate radial growth. Measurements were taken along two perpendicular axes, and mean values were calculated to improve accuracy. Microscopic observations were performed using a Leica Galen III™ phase-contrast light microscope (Leica Microsystems, Germany) equipped with an HDCE-50B digital camera, asexual structures, including conidiophores (sporophores), phialides, and conidia, were examined. For each isolate, 20 conidiophores and 20 conidia were measured to determine size (length, width, and septation). Structures were randomly selected to minimize observational bias. The arrangement and density of conidiophores, as well as the number of conidia per conidiophore, were also recorded. Micrographs were captured using a Canon EOS 2000D DSLR camera (Canon Inc., Japan) and an iPhone 14 Pro Max (Apple Inc., USA).

Morphological and morphometric data were compared with taxonomic descriptions from the literature, including Frisvad and Samson^30^, Pitt and Hocking^15^, Samson et al.^31^, Tashiro et al.^19^, and^32^, to support preliminary species identification. All isolates exhibited highly similar morphological characteristics, indicating low phenotypic variability. Considering this homogeneity, together with the low incidence of the disease observed across surveyed sites, two representative isolates (BH_B1 and BH_E2) from distinct origins (orchard and market) were selected for molecular analysis to ensure reliable species identification while avoiding unnecessary redundancy.

### PCR and molecular identification

The two representative isolates, BH_B1 and BH_E2, selected due to their morphological similarity and distinct origins (experimental station vs. market), were cultured on PDA at 25 °C for 7 days to obtain mature mycelium. Based on this observed homogeneity, two representative isolates were considered sufficient for molecular analysis to confirm species identity while avoiding redundancy. Genomic DNA was extracted using a modified cetyltrimethylammonium bromide (CTAB) protocol, originally described by Doyle and Doyle^33,34^ and subsequently adapted by Benzahra et al., (2026). DNA quality and integrity were assessed by electrophoresis on 1% agarose gel, and DNA concentration was determined using a NanoDrop™ spectrophotometer. The internal transcribed spacer (ITS) region and a fragment of the β-tubulin gene (BenA) were amplified using primer pairs ITS1 (5′-TCCGTAGGTGAACCTGCGG-3′) /ITS4 (5′-TCCTCCGCTTATTGATATGC-3′)^35^ and primers Bt2a (5′-GGTAACCAAATCGGTGCTGCTTTC-3′) / Bt2b (5′-ACCCTCAGTGTAGTGACCCTTGGC-3′)^36^. PCR reactions were performed in a final volume of 25 µL containing 1.5 µL of mi-Taq only 10× buffer, 0.2 mM of each dNTP, 0.4 µM of each primer, 0.6 U of mi-Taq only DNA polymerase (Invitrogen, Carlsbad, CA, USA), and approximately 50 ng of template DNA. The thermal cycling program for ITS amplification consisted of an initial denaturation at 95 °C for 5 min, followed by 35 cycles of 30 s at 95 °C, 30 s at 52 °C, and 1 min at 72 °C, and a final extension of 7 min at 72 °C. For β-tubulin amplification, the same program was applied, except that the annealing temperature was set at 58 °C. Amplified products were separated on 1% agarose gels in 1× TAE buffer, stained with ethidium bromide, and visualized using a Gel Doc system (Bio-Rad, USA). Fragment sizes were estimated using a 100 bp DNA ladder (New England Biolabs), and purified amplicons were sequenced bidirectionally by StabVida (Caparica, Portugal). The resulting sequences were deposited in the GenBank database, and accession numbers are provided in the Results section. Sequence identity was verified using BLAST analysis against the NCBI database, and closely related reference sequences were included in subsequent phylogenetic analyses.

### Pathogenicity of the fungus

Spore suspensions were prepared from isolates BH_B1 and BH_E2 and adjusted to a final concentration of 1 × 10⁶ conidia/mL using sterile distilled water. Pathogenicity assays were conducted on healthy, mature fruits of *Citrus sinensis*. The experiment was performed using three independent replicates, totaling 60 fruits, including inoculated and control groups, under controlled environmental conditions to ensure experimental consistency and reproducibility.

Prior to inoculation, fruits were surface-disinfected with 0.5% sodium hypochlorite, rinsed twice with sterile distilled water, and air-dried under aseptic conditions. Each fruit was wounded at the equatorial zone using a sterile needle (approximately 2–3 mm depth), and 10 µL of the conidial suspension was deposited into each wound. Control fruits were inoculated with sterile distilled water. Inoculated fruits were placed in sealed plastic containers lined with moistened paper towels to maintain high relative humidity (> 90%) and incubated at 25 ± 2 °C.

Symptom development was monitored daily for 7 days. Lesion diameter was measured at 2, 4, 6, and 7 days post-inoculation using a digital caliper, and mean values along with standard deviations were calculated. To fulfill Koch’s postulates, the pathogen was re-isolated from symptomatic tissues. Small fragments excised from lesion margins were cultured on PDA, and the identity of the re-isolated fungus was confirmed by morphological comparison with the original isolates.

### Phylogenetic analyses

Sequences obtained from the ITS and β-tubulin regions were edited and assembled using BioEdit v.7.2.5, and aligned with reference sequences retrieved from the GenBank database using MEGA11^37^.

The final concatenated alignment, including ITS and BenA sequences, was used for phylogenetic analyses, and details of all sequences included in the study are provided in Supplementary Table S1. Phylogenetic analyses were performed using Maximum Likelihood (ML) and Maximum Parsimony (MP) methods. The best-fit nucleotide substitution model (T92 + G) was selected based on the lowest Bayesian Information Criterion (BIC) value. The robustness of the phylogenetic trees was evaluated using bootstrap analysis with 1000 replicates. Only bootstrap values ≥ 50% were considered significant and are indicated at the nodes. Reference sequences representing closely related *Penicillium* species were selected from GenBank to ensure accurate phylogenetic placement of the studied isolates. *Penicillium atramentosum* (E371) was used as the outgroup to root the phylogenetic trees.

## Results and discussion

### Symptoms

The disease was observed on fruits of *Citrus sinensis* and *Citrus × limon* collected from both orchard and market environments, representing different stages of the postharvest chain. During surveys conducted between February 2024 and June 2025 across 36 sites (30 orchards and 6 local markets), atypical symptoms consistent with *Penicillium ulaiense* infection were detected at only two locations: one experimental orchard (INRA El Menzeh station, Kénitra) and one local market. This restricted distribution indicates that the occurrence of the disease was rare under the conditions surveyed. In the affected orchard, 25 trees were examined, with 10 fruits per tree (250 fruits in total), among which only four fruits were infected, corresponding to an incidence of 1.6%. At the market level, 200 fruits were inspected, and only one fruit showed similar symptoms (0.5%). No comparable symptoms were observed in the remaining sites surveyed. These observations indicate a low level of disease occurrence. However, given the limited number of infected samples and positive sites, these findings should be considered preliminary and not representative of the actual prevalence of the pathogen in Morocco.

**Fig. 1:**
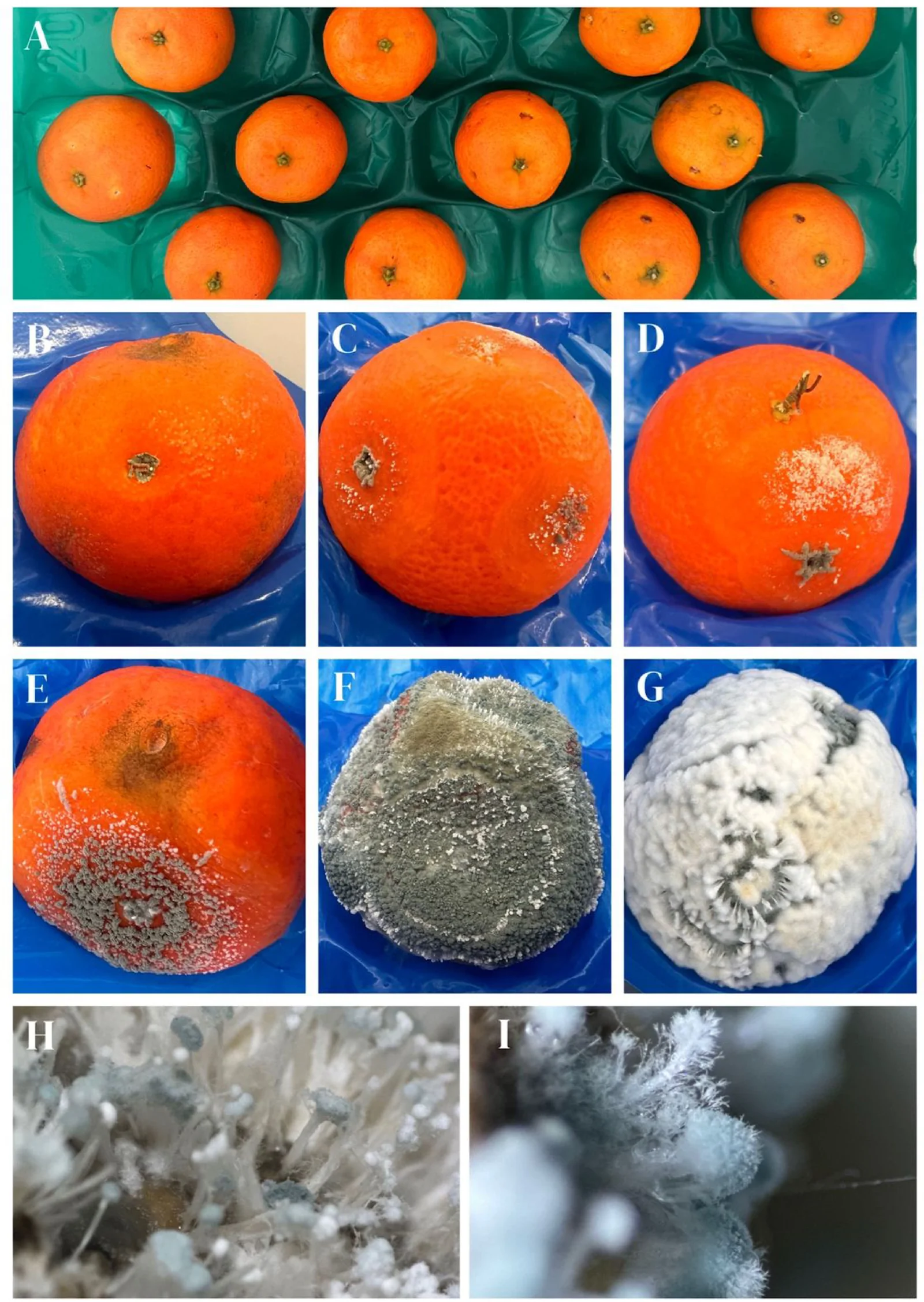
Progression of disease symptoms following wound inoculation with *Penicillium ulaiense*: (**A**) Asymptomatic inoculated fruit at day 0 showing no visible symptoms; (**B**) small, circular, water-soaked lesion developing at the inoculation site after 48 h; (**C**,**D**) lesion enlargement with tissue softening, white mycelial growth, and initial sporulation after 5 days; (**E**) advanced decay characterized by extensive tissue maceration and abundant blue-gray conidial masses after 10 days; (**F**) severe fruit rot with widespread fungal colonization and the formation of well-developed, erect, and morphologically distinctive coremia after 14 days; (**G**) late stage of infection showing complete fruit collapse, dense aerial mycelium, and numerous prominent coremia bearing compact conidial masses after 20 days. (**H**) close-up view of individual coremia, showing erect, compact structures composed of aggregated conidiophores with dense conidial masses at the apex; (**I**) detailed view of mature coremia, highlighting their elongated morphology, compact conidial heads, and abundant conidia, a characteristic feature of *P. ulaiense*.

Initial symptoms appeared as small, circular to slightly irregular, light brown lesions with a water-soaked appearance and poorly defined margins, generally occurring at wound sites (Fig. 1A). Lesions progressively expanded both radially and in depth, becoming more evident as tissue maceration advanced within the flavedo and albedo layers, reaching an average diameter of 5.2 ± 1.1 mm at 24 h (*n* = 5 fruits). A thin white mycelial mat developed at the lesion center during early colonization (Fig. 1B).

As the infection progressed, lesions exhibited a well-defined zonation pattern, characterized by abundant blue-gray powdery conidial masses in the central region and a distinct white peripheral halo (Fig. 1C). The halo width (2–3 mm) represents a useful diagnostic feature. Compared with *P. digitatum*, which produces homogeneous olive-green sporulation with a narrow margin, and *P. italicum*, which causes wet lesions with intense blue sporulation but lacks clear zonation, the symptoms observed here show an intermediate halo width combined with distinct zonation, providing a reliable criterion for distinguishing *P. ulaiense*.

At advanced stages, lesions became deeply sunken and softened, reflecting extensive tissue degradation (Fig. 1D,E), with coremia-like structures predominantly developing at the lesion periphery, particularly at the interface between the white marginal zone and the sporulating center. (Fig. 1C,F). These structures appeared 2–3 days after symptom onset under high humidity conditions (> 90%), consistent with the experimental conditions used in pathogenicity assays. They reached 3–5 mm in length and were clearly visible, markedly longer and more characteristic than those typically reported for *P. italicum*. Consequently, lesions exhibited a clear spatial contrast between centrally concentrated powdery sporulation and peripheral tuft-like coremia formation (Fig. 1F), a highly distinctive feature of *P. ulaiense* infections. 

At the final stages, affected tissues evolved into complete soft rot, characterized by total loss of firmness and structural integrity, followed by fruit shriveling, dehydration, and eventual mummification (Fig. 1D,E). Such severely colonized fruits may constitute an important source of secondary inoculum during postharvest handling and storage. 

Overall, the symptoms described in the present study are consistent with previous descriptions of *P. ulaiense* reported in the literature, while clearly differing from those typically associated with *P. digitatum* and *P. italicum*^15^^, 29^^, 31,38^. Several authors have emphasized that infections caused by *P. ulaiense* are typically characterized by pronounced lesion zonation, abundant blue-gray central sporulation, a distinct white peripheral margin, and the frequent development of coremia-like structures, considered a key diagnostic feature^15^^, 18^^, 30,31,39,40^. In contrast, green mold caused by *P. digitatum* generally produces more homogeneous lesions, olive-green to bright green in color, with a narrow peripheral margin and without coremia development, whereas blue mold induced by *P. italicum* is characterized by pronounced tissue depression and intense blue sporulation, also lacking comparable erect structures^15,38,41,29^. Thus, the combination of an intermediate white peripheral margin, abundant powdery central sporulation, and well-developed coremia provides *P. ulaiense* with a distinct symptom profile that is particularly useful for the differential diagnosis of postharvest citrus diseases.

### Culture characteristics and morphology of the fungus on PDA

**Fig. 2:**
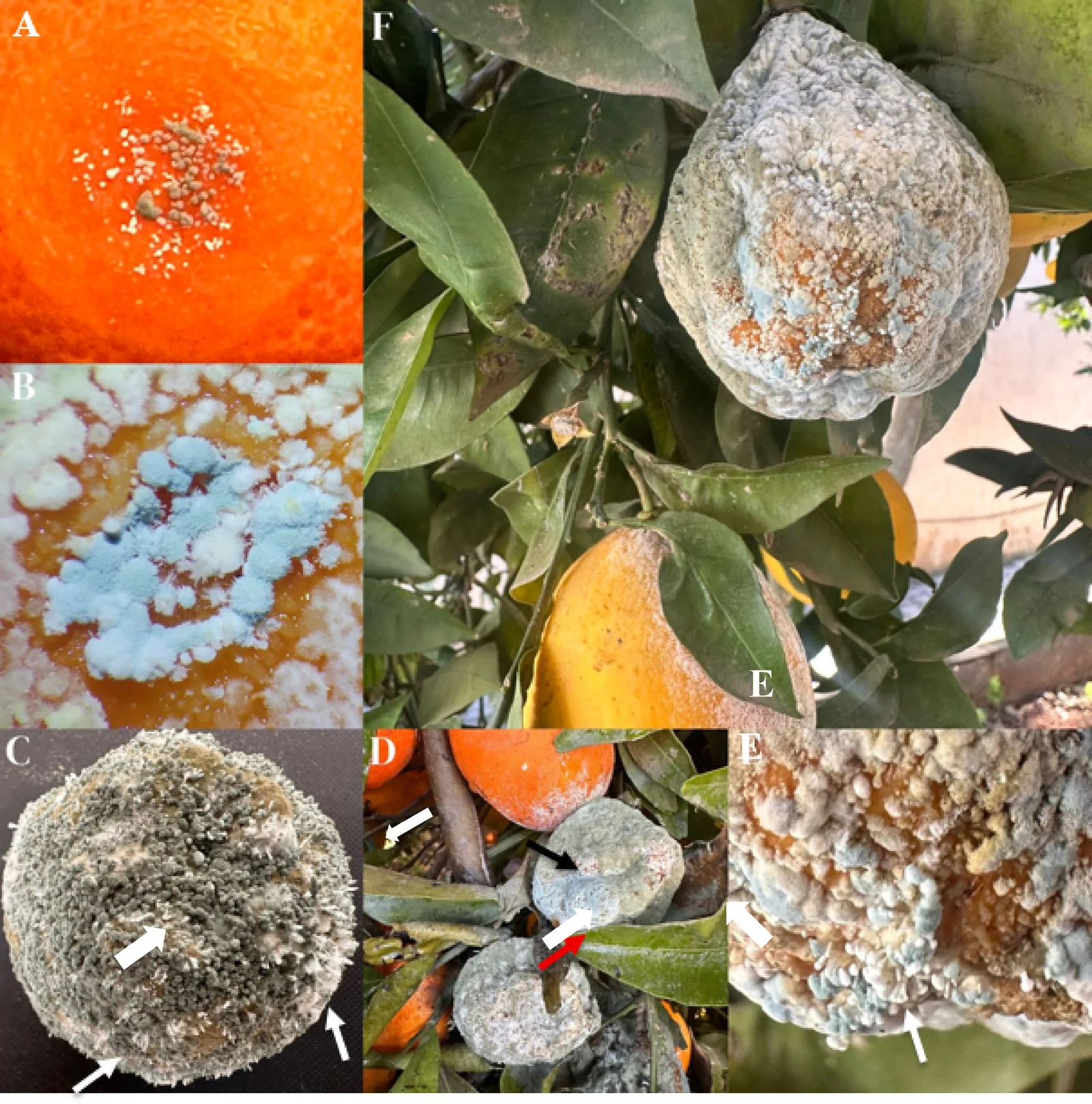
Natural disease symptoms observed on citrus fruits infected by *Penicillium ulaiense*. (**A**) Early stage of infection observed 24 h after symptom onset, showing small necrotic lesions with initial sporulation. (**B**) Disease progression 48 h after symptom onset, characterized by lesion expansion and increased bluish-gray sporulation. (**C**) Advanced fruit decay with extensive colonization of the fruit surface and dense sporulation. (**D**) Mixed mold infection on citrus fruit showing the association of whisker mold (*P. ulaiense*) with other *Penicillium* species; the black arrow indicates *P. digitatum*, the red arrow indicates *P. italicum*, and the white arrow highlights the well-developed coremia (whisker-like structures) characteristic of *P. ulaiense.* (**E**) Severely infected fruit remaining attached to the tree, completely covered by fungal mycelium and sporulating structures. (**F**) Close-up view of the fruit surface showing numerous erect, compact coremia bearing dense conidial masses, a distinctive morphological feature of *P. ulaiense* under natural infection conditions.

After 7 days of incubation on Potato Dextrose Agar (PDA) at 25 ± 2 °C, the fungal isolates (BH_B1, BH_E2, BH_M3, BH_A4, BH_G5) developed circular, slow-growing colonies with a dull blue-gray to gray-green surface, a distinct white marginal zone, and a yellow to orange pigmentation on the reverse (Fig. 2A,B). A radial zonation pattern was evident, accompanied by abundant aerial mycelium. Short upright stalks corresponding to coremia emerged from the colony surface (Fig. 2C). These characteristics were consistently observed across independent replicates (*n* = 3 per isolate), confirming the reproducibility of the observations.

All isolates produced well-developed aerial conidiomata, initially short and erect, which elongated to form mature structures bearing dense conidial masses at their apex, reaching 1–8 mm in height (Fig. 2D,E). These conidiomata represent a distinctive macroscopic feature of *Penicillium ulaiense*, consistent with the “whisker mold” reported in Japan and California^40,42^.

**Fig. 3:**
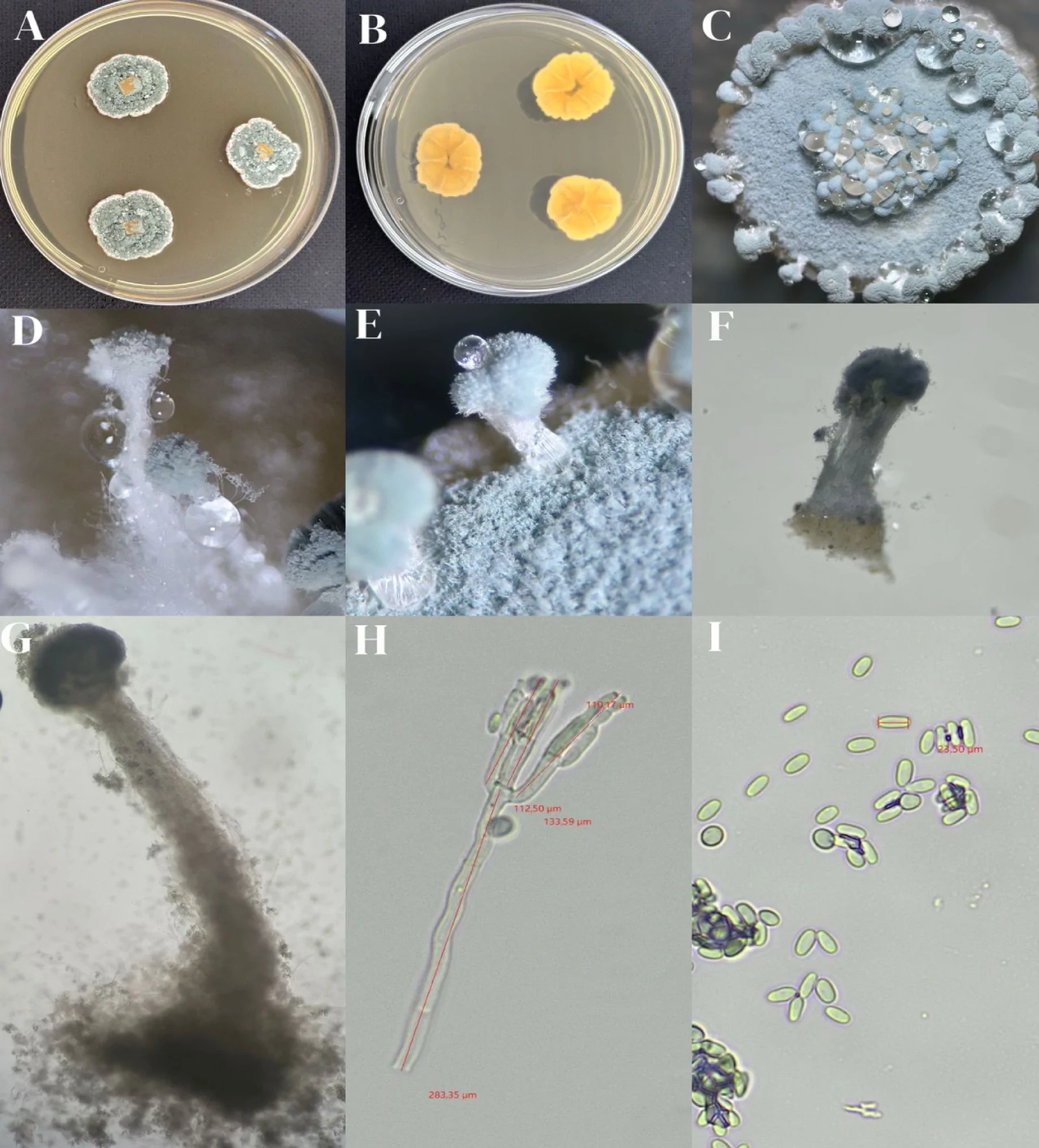
Morphological and cultural characteristics of the fungal isolate of *Penicillium ulaiense*. (**A**) Colony morphology of the fungal isolate grown on potato dextrose agar (PDA) after 7 days of incubation at 25 ± 2 °C, showing circular colonies with a bluish-gray surface and a white marginal zone; (**B**) Reverse side of colonies on PDA, exhibiting a yellow to orange pigmentation at the colony center; (**C**) Close-up view of the colony surface, showing abundant aerial mycelium and the formation of erect conidiomatal structures; (**D**) Early development of coremia-like structures emerging from the mycelial mat; (**E**) Mature coremia-like structure bearing a dense conidial mass at the apex; (**F**) Light microscopic view of a single erect conidiophore forming a compact conidial head; (**G**) Detailed microscopic observation of the conidiophore and terminal conidial mass; (**H**) Conidiophores with phialides producing conidia; measurements correspond to typical dimensions of the conidiogenous structures; (**I**) Ellipsoidal to ovoid, hyaline conidia observed under light microscopy, occurring singly or in small aggregates.

Microscopic examination confirmed that these upright structures corresponded to aggregated conidiophores forming compact conidial heads (Fig. 2F,G). The penicilli were predominantly ter verticillate, irregularly branched, and often divergent (Fig. 2H). In the examined isolates, each penicillus had 2–3 branches (65.73–82.90 × 3.0–4.5 μm). The metulae, smooth-walled, measured 48–59 × 3.0–4.5 μm and were arranged in verticils of 2–5, each bearing 3–5 densely packed phialides (20.6–40.5 × 3.5–4.5 μm). The conidia, primarily cylindrical to sub-ellipsoidal and smooth-walled, exhibited limited size variation, ranging from 4.0 to 13.0 × 2.5–3.8 μm (Fig. 3; Table 1). All measurements were based on observations of at least 20 conidiophores and 20 conidia per isolate, randomly selected to reduce observational bias. Although slightly larger than those reported by Tashiro et al.^19^ for Japanese isolates (4–11 × 2–4.5 μm), these dimensions remain within the known morphological variability for this species, and such variation is likely attributable to differences in culture medium, incubation conditions, and geographic origin of the isolates rather than taxonomic divergence.

**Table 1:** GenBank accession numbers, sampling site coordinates, conidial dimensions, colony growth on potato dextrose agar (PDA), and coremia size of *Penicillium ulaiense* isolates obtained from citrus fruits in the present study between February 2024 and June 2025. Colony growth values correspond to the sum of horizontal and vertical measurements recorded after incubation.

Isolate	Origin site	Geographic coordinates	GenBank accession (ITS)	GenBank accession (β-tubulin)	Conidial size (µm) Min–Max (L × W)	Conidial size (µm) Mean ± SD (L × W)	L/W ratio	Colony size on PDA (mm) Min–Max	Colony size on PDA (mm) Mean ± SD	Coremia size (mm) Min–Max	Coremia size (mm) Mean ± SD
BH_B1	INRA El Menzeh Station	34.2935507, -6.4862050	PV822027	PX095996	(4.0–11.8) × (2.6–3.6)	5.2 ± 1.6 × 3.1 ± 0.3	1.68	15.0–18.0	15.8 ± 2.1	2.5–5.0	3.9 ± 0.8
BH_E2	Local markets	34.2620605, -6.5622416	PV822028	PX725984	(4.2–13.0) × (2.5–3.8)	5.9 ± 2.1 × 3.0 ± 0.4	1.97	12.5–19.0	16.2 ± 1.9	2.8–6.0	4.3 ± 1.0
BH_M3	INRA El Menzeh Station	34.2935507, -6.4862050	-	-	(4.0–12.5) × (2.7–3.5)	5.6 ± 1.9 × 3.2 ± 0.3	1.75	10.0–17.5	12.9 ± 2.3	3.5–8.0	6.1 ± 1.4
BH_A4	Local markets	34.2620605, -6.5622416	-	-	(4.1–10.9) × (2.8–3.6)	5.0 ± 1.4 × 3.3 ± 0.3	1.52	13.0–20.5	16.6 ± 1.5	2.6–7.5	5.05 ± 0.9
BH_G5	INRA El Menzeh Station	34.2935507, -6.4862050	-	-	(4.3–12.7) × (2.6–3.7)	5.8 ± 2.0 × 3.1 ± 0.4	1.87	11.5–16.8	13.7 ± 2.0	2.2–6.0	3.6 ± 0.7

Data are expressed as minimum–maximum values and mean ± standard deviation (SD) based on nine replicates per isolate.

Conidial dimensions are expressed as length × width (µm).

L/W ratio was calculated from mean conidial length and width.

Colony size and coremia size are expressed in millimeters (mm).

“–” indicates isolates that were not sequenced.

For differential diagnosis, our isolates were compared with other citrus-pathogenic species such as *P. italicum* and *P. digitatum*. Compared with *P. italicum*, the isolates did not exhibit intense blue sporulation lacking conidiomata, and compared with *P. digitatum*, colonies showed less compact mycelial growth and a duller coloration. The presence of well-developed conidiomata, bluish-gray sporulation, and characteristic penicillus morphology provide reliable macroscopic and micromorphological criteria to distinguish *P. ulaiense* from closely related species.

Overall, the cultural and micromorphological observations closely match previous descriptions of *P. ulaiense*^30,40,43^ and are consistent with Japanese isolates studied by Tashiro et al.^19^. However, given the limited number of isolates examined (*n* = 5), these observations should be interpreted as representative rather than exhaustive of the morphological variability of the species. Accordingly, the present results support the identification of the isolates as *P. ulaiense* and provide the first detailed morphological description of this species on citrus fruits in Morocco, while remaining cautious regarding broader generalizations.

### Molecular identification

The ITS and β-tubulin sequences of the Moroccan *P. ulaiense* isolates (BH_B1 and BH_E2), deposited in GenBank under accession numbers PV822027/PX095996 and PV822028/PX725984, were compared with international reference isolates. The selected isolates originated from distinct sampling contexts and were chosen for their morphological representativeness, allowing coverage of the observed variability while minimizing redundancy. Multiple sequence alignment of ITS and β-tubulin regions revealed a high level of sequence similarity among the analyzed isolates, with only limited polymorphisms, including nucleotide substitutions and small insertions/deletions. The final concatenated alignment (ITS + β-tubulin) comprised 804 base pairs after alignment and careful manual refinement. These limited polymorphisms enabled clear distinction of *P. ulaiense* from closely related species, notably *P. italicum* and *P. expansum*, with no overlap in diagnostic sequence positions between species, while indicating low intraspecific polymorphism despite the geographically diverse origins of the isolates studied^30,42^.

BLAST analyses further confirmed the identity of the Moroccan isolates. Isolate BH_B1 showed 100% similarity in the β-tubulin region with several *P. ulaiense* isolates (MH477873.1, MH257623.1, PX095996.1) and 99% with other reference isolates (PV058983.1, PV058984.1), while ITS sequences exhibited > 99% identity with reference sequences (AB674741.1, OW984294.1, MH575188.1). Similarly, isolate BH_E2 displayed 100% identity in β-tubulin region with multiple isolates (AB671774.1, AY674406.1, MH780068.1, LT629297.1, MH477873.1, PX725984), with ITS identity also exceeding 99%, whereas similarity to more divergent isolates was lower. These results provide strong support for the assignment of the Moroccan isolates to *P. ulaiense* and indicate low observed genetic variability within the available dataset, without implying geographic uniformity. The reference sequences used for BLAST comparison and phylogenetic reconstruction are provided in Supplementary Table S1, ensuring transparency and reproducibility of the analyses^17,42^.

**Fig. 4:**
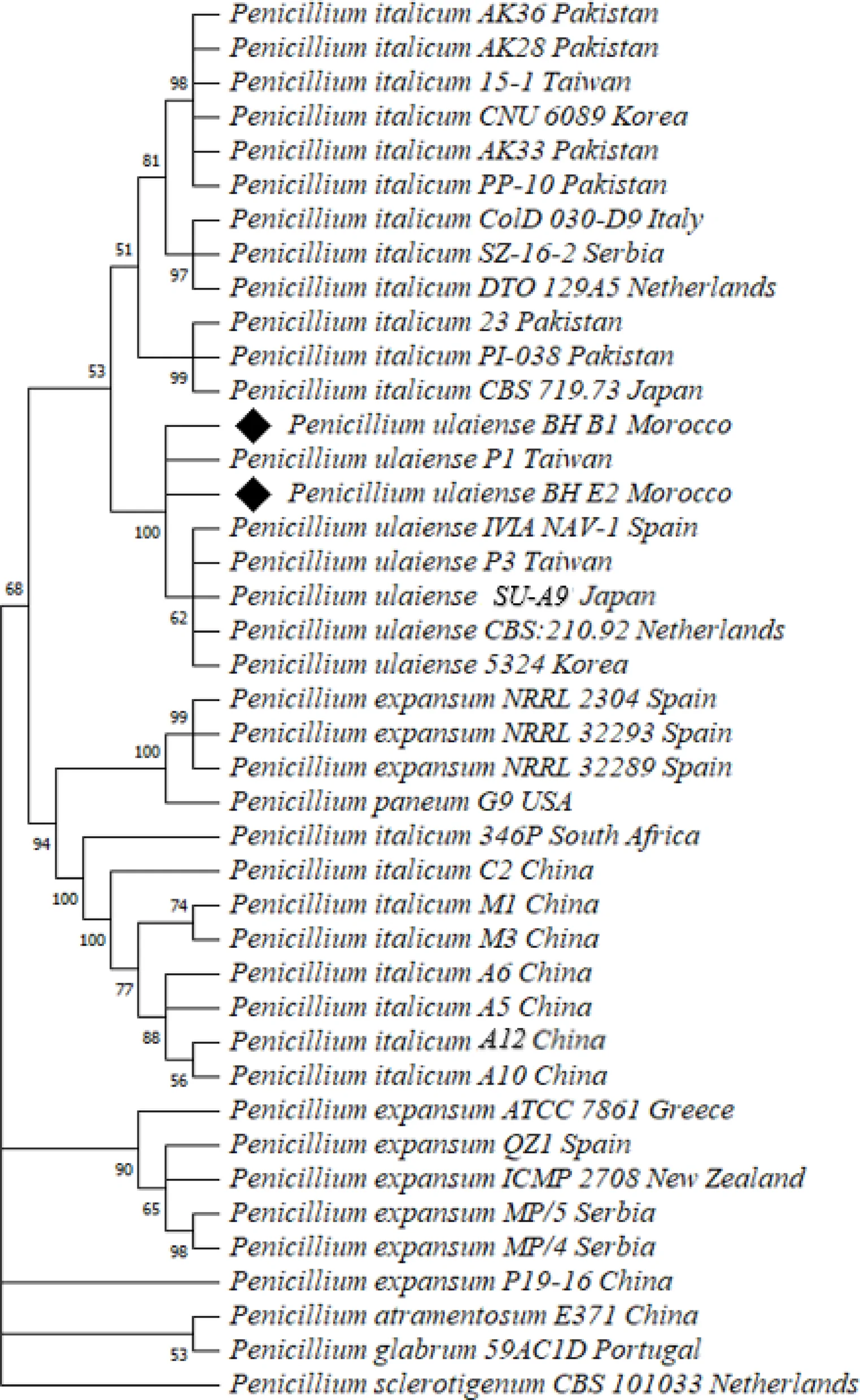
Phylogenetic tree reconstructed from maximum likelihood (ML) and maximum parsimony (MP) analyses of concatenated ITS and β-tubulin sequences. The ML analysis was performed using the Tamura three-parameter model (+ G) with 1000 bootstrap replicates. Bootstrap values above 50% are indicated at the nodes for both ML and MP analyses. The Moroccan isolates BH_B1 and BH_E2 obtained in this study are highlighted by a black diamond (♦). *Penicillium glabrum* (59AC1D) and *Penicillium sclerotigenum* (CBS 101033) were used as outgroup taxa. The scale bar represents the number of nucleotide substitutions per site.

Phylogenetic analysis based on concatenated ITS and β-tubulin sequences showed that the Moroccan isolates clustered within a well-supported monophyletic clade of *P. ulaiense* (Fig. 4), together with isolates from Taiwan, Spain, Japan, the Netherlands, and Korea, with high bootstrap support (> 95%), confirming their taxonomic placement. This clustering was consistently supported by both Maximum Likelihood and Maximum Parsimony approaches. *P. italicum* isolates were distributed among several subclades reflecting their geographic origin (Pakistan, China, Taiwan, Korea, Italy, Serbia, the Netherlands, Japan), whereas *P. expansum* formed a distinct clade including isolates from Spain, Greece, New Zealand, and Serbia, illustrating clear species-level divergence. The inclusion of internal (*P. paneum*) and external (*P. glabrum* and *P. sclerotigenum*) outgroups contributed to tree stability and supported the monophyly of the analyzed clades. These findings are consistent with the morphological identification and further support the use of an integrative approach combining morphological and molecular data^30,42^. The clustering of Moroccan isolates with international strains reflects the limited variability observed within the available sequence dataset and should be interpreted with caution, given the still limited global sampling of this species. The clear separation between *P. ulaiense*, *P. italicum*, and *P. expansum* confirms the reliability of ITS and β-tubulin markers for discriminating closely related species^17,36,42^.

However, considering the limited number of isolates analyzed (*n* = 2 for molecular data), these findings should be interpreted as representative rather than exhaustive. Overall, the results provide a reliable basis for the molecular identification of *P. ulaiense* in Morocco, while highlighting the need for further studies incorporating a larger number of isolates and additional genetic markers.

### Pathogenicity test

Pathogenicity assays confirmed that both isolates of *P. ulaiense* (BH-B1 and BH-E2) were pathogenic to *Citrus sinensis* fruits. Typical symptoms developed at inoculation sites within 2 days post-inoculation and were monitored over a 7-day period (Fig. 1B–I). Initially, lesions appeared as small, circular, light-brown, water-soaked spots measuring approximately 2–3 mm in diameter (Fig. 1B). Lesion development was quantitatively assessed, showing a progressive increase in size, reaching 8–12 mm in diameter by the end of the experiment, reflecting tissue maceration. As infection progressed, infected fruits exhibited characteristic soft rot symptoms, accompanied by the formation of a dense white mycelial mat at the center of the lesions (Fig. 1C,D). Subsequently, abundant bluish-gray conidial masses developed on the lesion surface under high-humidity conditions (Fig. 1E). All inoculated fruits (*n* = 20 per isolate) developed symptoms consistently across independent replicates, whereas control fruits remained asymptomatic throughout the experiment (Fig. 1A).

At advanced stages of infection, lesions became deeply sunken and extended beyond the original wound margins. In several cases, coremia-like structures typical of *P. ulaiense* were observed on the surface of infected tissues (Fig. 1F–I). The pathogen was successfully re-isolated from symptomatic tissues in all cases, and the recovered isolates exhibited morphological characteristics identical to the original cultures. These results confirm the pathogenicity of the isolates and fulfill Koch’s postulates, while remaining consistent with the controlled experimental conditions described.

## Conclusion

This study provides the first report of *Penicillium ulaiense* associated with postharvest whisker mold of citrus fruits in Morocco. The integrative approach adopted, combining morphological characterization, multilocus molecular identification (ITS and β-tubulin), phylogenetic analyses, and pathogenicity assays, allowed a robust confirmation of both the identity of the pathogen and its involvement in symptom development under the experimental conditions of this study. The characteristic symptoms, particularly lesion zonation and the formation of coremia-like structures, constitute reliable and discriminative diagnostic features that clearly distinguish *P. ulaiense* from other major postharvest citrus pathogens. Given the limited number of samples and isolates analyzed, this work should be considered a preliminary report, and the results cannot be extrapolated to the broader citrus production systems in Morocco. The low level of occurrence and incidence observed across the surveyed sites suggests that this pathogen was detected only sporadically under the conditions of this work. Thus, these findings provide the first evidence of the presence of *P. ulaiense* in Morocco and establish a solid scientific basis for future large-scale investigations. Further studies will be necessary to better characterize its distribution, prevalence, and potential impact in citrus production systems, as well as to clarify its significance within the context of postharvest diseases.

## Supplementary Information

Below is the link to the electronic supplementary material.

Supplementary Material 1

## Data Availability

The ITS and β-tubulin (BenA) sequences generated in this study have been deposited in GenBank under the following accession numbers: PV822027 (ITS, isolate BH_B1), PX095996 (BenA, isolate BH_B1), PV822028 (ITS, isolate BH_E2), and PX725984 (BenA, isolate BH_E2). All other data supporting the findings of this study are available from the corresponding author upon reasonable request.
