# Supplementary material for: First report of Penicillium ulaiense causing whisker mold on postharvest citrus fruits in Morocco
Source: Sci Rep. 2026 Jun 22;16:28692. doi: 10.1038/s41598-026-56301-2 (PMC13575172; doi:10.1038/s41598-026-56301-2)
Supplement: Supplementary file 1 [file 41598_2026_56301_MOESM1_ESM.docx]

**Supplementary Table 1.** GenBank accession numbers and morphological characteristics of *Penicillium ulaiense* isolates obtained from citrus fruits in Morocco and reference sequences used in this study.

| Species | Isolate | Origin | NCBI GenBank Accession Number | | References |
| --- | --- | --- | --- | --- | --- |
|  |  |  | ITS | *TUB2* |  |
| *Penicillium italicum* | AK36 | Pakistan | MK736928 | MK947453 | - |
| *Penicillium italicum* | AK28 | Pakistan | MK736920 | MK947447 | - |
| *Penicillium italicum* | 15-1 | Taiwan | PX373084 | QBK46502 |  |
| *Penicillium italicum* | CNU 6089 | Korea | HQ225716 | HQ225728 | - |
| *Penicillium italicum* | AK33 | Pakistan | MK947451 | MK736925 | - |
| *Penicillium italicum* | PP-10 | Pakistan | PQ009242 | OM161189 | - |
| *Penicillium italicum* | ColD_030-D9 | Italy | OR361397 | XBG96676 |  |
| *Penicillium italicum* | SZ-16-2 | Serbia | OR633248 | OR644439 | - |
| *Penicillium italicum* | DTO 129A5 | Netherlands | KJ775641 | KJ775134 | - |
| *Penicillium italicum* | 23 | Pakistan | MZ351316 | ON082768 | - |
| *Penicillium italicum* | PI-038 | Pakistan | OP186023 | MT573505 |  |
| *Penicillium italicum* | CBS 719.73 | Japan | KC411773 | PV058193 |  |
| *Penicillium ulaiense* | BH B1 | Morocco | PV822027 | PX095996 | The present study |
| *Penicillium ulaiense* | P1 | Taiwan | MH887434 | MH899764 |  |
| *Penicillium ulaiense* | BH E2 | Morocco | PV822028 | PX725984 | The present study |
| *Penicillium ulaiense* | IVLA NAV-1 | Spain | MH477872 | MH477873 |  |
| *Penicillium ulaiense* | P3 | Taiwan | MH887435 | MH899765 | - |
| *Penicillium ulaiense* | [SU-A9](https://www.ncbi.nlm.nih.gov/nuccore/AB671774.1) | Japan | AB671774 | AB674741 | - |
| *Penicillium ulaiense* | CBS:210.92 | Netherlands | KC411695 | AY674408 | - |
| *Penicillium ulaiense* | 5324 | Korea | KJ527466 | KJ527431 | Park et al., 2014 |
| *Penicillium expansum* | NRRL 2304 | Spain | DQ339556 | FJ457086 | - |
| *Penicillium expansum* | NRRL 32293 | Spain | DQ339552 | FJ457085 |  |
| *Penicillium expansum* | NRRL 32289 | Spain | DQ339547 | FJ457084 | - |
| *Penicillium paneum* | G9 | USA | KX243325 | JX127316 | - |
| *Penicillium italicum* | 346P | South Africa | EU128647 | EU128589 | - |
| *Penicillium italicum* | C2 | China | HQ850920 | HQ850954 | - |
| *Penicillium italicum* | M1 | China | HQ850931 | HQ850963 | - |
| *Penicillium italicum* | M3 | China | HQ850930 | HQ850965 | - |
| *Penicillium italicum* | A6 | China | HQ850905 | HQ850939 | - |
| *Penicillium italicum* | A5 | China | HQ850904 | HQ850938 | - |
| *Penicillium italicum* | A12 | China | HQ850904 | HQ850911 | - |
| *Penicillium italicum* | A10 | China | HQ850909 | HQ850943 | - |
| *Penicillium expansum* | ATCC 7861 | Greece | AY373912 | NR_077154 | - |
| *Penicillium expansum* | QZI | Spain | MK660357 | MK675788 | Ramos-Pereira et al., 2019 |
| *Penicillium expansum* | ICMP 2708 | New Zealand | MZ393463 | MW862779 | Weir et., 2025 |
| *Penicillium expansum* | MP/4 | Serbia | MT563326 | MT556009 | - |
| *Penicillium expansum* | P19-16 | China | GU723453 | PQ787362 | - |
| *Penicillium atramentosum* | E371 | China | OQ257204 | PQ877029 | - |
| *Penicillium glabrum* | 59ACID | Portugal | GU372902 | GU372882 | Barreto et al., 2011 |
| *Penicillium sclerotigenum* | CBS 101033 | Netherlands | MH862718 | AY674393 | - |

*“–“ Sequences of Moroccan isolates were generated in the present study and are available in GenBank via direct submission, with no associated published references.*

**References**

1. Barreto, M. C., Houbraken, J., Samson, R. A., Frisvad, J. C. & San-Romão, M. V. Taxonomic studies of the Penicillium glabrum complex and the description of a new species P. subericola. Mycol. Prog. 10, 449–460 (2011).

2. Park, M. S., Fong, J. J., Oh, S. Y., Kwon, K. K., Sohn, J. H. & Lim, Y. W. Marine-derived Penicillium in Korea: diversity, enzyme activity, and antifungal properties. Antonie Van Leeuwenhoek 106, 331–345 (2014).

3. Ramos-Pereira, J., Mareze, J., Patrinou, E., Santos, J. A. & López-Díaz, T.-M. Polyphasic identification of Penicillium spp. isolated from Spanish semi-hard ripened cheeses. Food Microbiol. 84, 103253 (2019).

4. Weir, B. S. et al. Global diversity analysis of plant-associated Pseudopithomyces fungi reveals a new species producing the toxin associated with facial eczema in livestock: Pseudopithomyces toxicarius sp. nov. Stud. Mycol. 112, 39–73 (2025).
